# Hereditary breast cancer and ancestry in the Madeira archipelago: an exploratory study

**DOI:** 10.3332/ecancer.2021.1261

**Published:** 2021-07-05

**Authors:** Isália Miguel, Fátima Rodrigues, Sofia Fragoso, João Freixo, Ana Clara, Ana Luís, Sandra Bento, Mariana Fernandes, Filipe Bacelar, Sara Câmara, Joana Parreira, Teresa Duarte, Paula Rodrigues, Sidónia Santos, Fátima Vaz

**Affiliations:** 1 Instituto Português de Oncologia de Lisboa Francisco Gentil, EPE, Rua Prof. Lima Basto 1099-023 Lisboa, Portugal; 2 CGPP-IBMC-i3S - Centro de Genética Preditiva e Preventiva, Instituto de Biologia Molecular e Celular, Instituto de Investigação e Inovação em Saúde, Universidade do Porto, Rua Júlio Amaral de Carvalho 45, 4200-135 Porto, Portugal; 3 Hospital Dr Nélio Mendonça, SESARAM, EPE, Avenida Luís de Camões 57, 9004-514 Funchal, Portugal

**Keywords:** hereditary breast and ovarian cancer syndrome, founder effect, genetic testing, pathogenic variant

## Abstract

Access to genetic testing and counselling in remote areas such as the Madeira archipelago, in the Northern Atlantic Ocean, may be complex. Different counselling methods, including telegenetics, should be explored. In this study, we characterise the Hereditary Breast/Ovarian Cancer (HBOC) families with Madeira ancestry enrolled in our programme. Of a total of 3,566 index patients tested between January 2000 and June 2018, 68 had Madeira ancestry and 22 were diagnosed with a pathogenic germline variant (PV). As in the whole group, BRCA2 PV were more frequent in Madeira patients (68.4%: c.9382C>T (26.3%), c.658_659del (21%), c.156_157insAlu (10.5%), c.793+1G>A (5.3%) and c.298A>T (5.3%). However, the most frequently diagnosed PV in Madeira patients was the BRCA1 c.3331_3334del (31.6%). *BRCA1/2* detection rates were 27.9% and 10.5% for Madeira and the whole group, respectively. This study is the first characterisation of HBOC patients with Madeira ancestry. A distinct pattern of *BRCA1/2* variants was observed, and the geographic clustering of *BRCA1* c.3331_3334del variant may support the possibility of a founder mutation previously described in Northern Portugal. The high detection rate observed reinforces the need to reduce gaps in access to genetic testing in Madeira and other remote areas. According to current guidelines, timely identification of HBOC patients can contribute to their ongoing care and treatment.

## Background

The identification of pathogenic *BRCA1* and *BRCA2* variants has had a marked impact on cancer prevention and therapy, with criteria for *BRCA1*/2 testing being included in several cancer treatment and prevention guidelines [1–3]. Thus, the expansion of recommendations for *BRCA1*/2 testing has challenged established delivery models of care for access to genetic counselling and testing [1, 4]. This access is even more complex in remote regions, where geographic limitations and lack of specialised programmes can negatively impact patient management and limit population-based genetic studies. Several models, including telegenetics, have been proposed to improve access to genetic testing without compromising quality of care [5]. These methods have not been found to be associated with long-term negative psychosocial outcomes [6].

In addition to targeted individual and family preventive and therapeutic management, knowledge of the geographical distribution of *BRCA1*/2 variants is an important tool for population-based studies. Indeed, it represents one of the most useful ways to approach genetic variation and penetrance estimation and to clarify genotype–phenotype correlations. *BRCA1* is described as the most frequently mutated gene in Hereditary Breast/Ovarian Cancer (HBOC) syndrome, but *BRCA2* variants seem to be more frequent in the Portuguese population as well as in women of East Asian and Icelandic ancestry [7–9]. An excess of specific variants in certain populations has been associated with a founder effect, including in the Portuguese population, where *BRCA2* c.156_157insAlu accounts for more than one fourth of the identified *BRCA1*/2 families [7, 10, 11]. Furthermore, other *BRCA1* founder mutations have been described in HBOC families from North Portugal [7], but the distribution of *BRCA1*/2 variants is unknown in the Madeira archipelago. There may be carriers of as yet undiagnosed founder variants in these islands, which are located nearly 1,000 km from continental Portugal in the North Atlantic Ocean [9–10, 12]. We reviewed all consecutive HBOC families with Madeira ancestry registered in our programme, explored their *BRCA1*/2 genotyping results and compared them with our data from continental Portugal.

## Methods

### Ethics approval

All procedures and consent forms were approved by the local Ethics Committee and all patients or their legal representatives signed an informed consent form.

### Patients & methods

In January 2000, a multidisciplinary HBOC programme was implemented in the Instituto Português de Oncologia de Lisboa. The objectives of this programme were the identification and risk management of HBOC patients. Initially, genetic testing was proposed for patients with a combined probability of at least 10% of having a *BRCA1/2* pathogenic variant (PV), but the criteria for testing have been expanded over time, including patients without a family history of cancer (e.g. non-mucinous ovarian cancer after 2014) [13]. Paternal and maternal ancestry is recorded, up to the third previous generation. Pre-test and post-test counselling was done in person or by telephone. As of June 2018, a total of 5,925 index patients were enrolled in the programme and 3,566 consented to genetic testing (*BRCA1/2*: 2,381 patients; multigene panel testing: 1,185 patients). The Madeira ancestry subgroup included 106 index patients (1.8%) and 68 of them consented to *BRCA1/2* testing, with four of them undergoing sequential *TP53* (2) or *PTEN* (2) analysis. Nine patients consented to upfront multigene testing.

The cancer diagnoses of the index patients were mostly breast (86%) and ovarian (9%) cancer, with 1% having both diagnoses. In the Madeira subgroup, 60 patients (88%) had breast cancer, six (8.8%) had ovarian cancer, and two (2.9%) had both. In this subgroup, eight patients had an additional cancer diagnosis (11.8%).

All patients were tested for *BRCA1/2* point variants, large rearrangements and the Portuguese founder variant in *BRCA2* c.156_157insAlu [11]. Before the introduction of next generation sequencing (NGS), heteroduplex-based methods such as Conformational Sensitive Gel Electrophoresis [14–15] and Conformational Sensitive Capillary Electrophoresis [14–16] were used. From 2014 to 2018, *BRCA1/2* point variants were analysed by NGS using the BRCA MASTR Dx kit (Multiplicom, Niel, Belgium) or multigene panels such as TruSight Cancer (Illumina) or the BRCA Hereditary Cancer MASTR Plus kit (Multiplicom, Niel, Belgium) in a MiSeq platform (Illumina, San Diego, CA, USA) according to the manufacturer’s protocol. Variant analysis was performed using DNAnexus software (CA, USA). All pathogenic or likely pathogenic variants were confirmed by Sanger sequencing. The genes tested in these panels included *BRCA1, BRCA2, PALB2, CHEK2, PTEN, CDH1, TP53, ATM, RAD51C, RAD51D, BRIP1, RAD50, BARD1, NBN* and *BLM*.

Large deletions/insertions in *BRCA1/2* genes were tested using Multiplex Ligation-dependent Probe Amplification (MRC-Holland, Amsterdam, The Netherlands) according to the manufacturer’s protocol in an ABI Prism 3130 Genetic Analyzer platform (Applied Biosystems, Foster city, USA) and analysed using Coffalyser.Net software (MRC-Holland, Amsterdam, The Netherlands). Variants were named according to Human Genome Variation Society nomenclature guidelines (version 15.11) and the reference sequences of *BRCA1/2, PALB2, ATM* and *RAD50* used are LRG_292t1, LRG_293t1, LRG_308t1, LRG_135t1 and NM _005732.3, respectively.

## Results

A total of 3,566 patients were tested exclusively for *BRCA1/2* (Table 1). Of these, 386 patients were diagnosed with a *BRCA1/2* PV (19 with Madeira ancestry). Regarding other genes, 62 out of 1,185 patients had non-*BRCA1/2* PV diagnosed, including 3 from the Madeira archipelago (Table 1). Detection rates, both for the entire programme and for the Madeira subgroup, were calculated considering only *BRCA1/2*-positive results and were higher for the Madeira subgroup (27.9% versus 10.5%).

Nineteen germline *BRCA1/2* pathogenic variants were identified in HBOC index patients from non-related families with Madeira ancestry (Table 1), with *BRCA2* being the most frequently mutated gene (68.4%). The c.3331_3334del in *BRCA1* (31.6%) and the c.9382C>T in *BRCA2* (26.3%) genes were the variants more recurrently identified. The c.3331_3334del BRCA1 was observed in six non-related families, being the only *BRCA1* pathogenic event in this subgroup. The *BRCA2* c.9382C>T, c.298A>T and c.658_659del variants were exclusively identified in patients with Madeira ancestry as well as the two PV in the *PALB2* gene (c.751C>T and c.1633G>T).

### Cascade testing

Within the 22 positive patients with a PV in the Madeira subgroup, a total of 59 family relatives were identified for cascade testing. All patients were given a detailed report so that local structures could identify additional relatives for testing and implementing risk-reducing measures in the case of carriers of pathogenic variants.

## Discussion

This study reports the first characterisation of genetic variants associated with HBOC in patients with Madeira ancestry. We observed a higher-than-expected mutation detection rate and a pattern of *BRCA1/2* variant distribution different from the rest of the country, although, as expected for Portuguese HBOC patients, *BRCA2* variants were diagnosed more frequently than *BRCA1* [7, 17].

Regarding the higher detection rate observed for Madeira patients, selection bias cannot be excluded, since most of our index patients lived or travelled frequently to the continent, thus having easy access to genetic programmes that were not available at the time in archipelago. However, this observation deserves further analysis as, if confirmed, it highlights the potential positive impact on cancer prevention and treatment outcomes for breast and ovarian cancer patients in the archipelago.

When comparing the pattern of *BRCA1/2* PV in the Madeira subgroup with that of the whole programme, a lower prevalence of the Portuguese *BRCA2* founder variant (c.156_157insAlu) was observed in Madeira (10.5% versus 23.4%). Of the three *BRCA2* variants only observed in Madeira patients (c.298A>T, c.658_659del and c.9382C>T), c.9382 C>T has been previously diagnosed in the North of Portugal [7].

The only *BRCA1* PV identified in the Madeira subgroup (c.3331_3334del) was previously proposed to be a founder variant of Northern Portuguese origin [7]. This genetic event has also been observed in other populations such as Brazil [18–20], Colombia [21], Spain [22] and Canada [23]. Our data could add to the possibility of a founder effect, since up to 54% of the first XVI century settlements in Madeira originated from Northern Portugal. Haplotype analysis could help determine the possibility of a common ancestor.

The patients included in this study have had access to genetic testing for more than a decade, and panels and testing methods have evolved over time. The implementation of NGS testing has allowed the identification of mutant genes other than *BRCA1/2* such as *PALB2*, *ATM* and *RAD50* [24, 25]. The *PALB2* variants (c.751C>T and c.1633G>T) were also exclusive to the Madeira subgroup, having previously been described in Portuguese, German, Russian and Chinese patients [26–28]. At this time, published evidence does not support the inclusion of *RAD50* in hereditary breast and ovarian multigene panels [29, 30].

## Conclusions

We observed a higher-than-expected detection rate and a distinct pattern of *BRCA1/2* variants in HBOC patients with Madeira ancestry. Confirmation of *BRCA1/2* variant distribution and further studies on geographic clustering of *BRCA1* c.3331_3334del will add to our knowledge of population genetics. The observed high detection rate highlights the need to reduce gaps in access to genetic testing in Madeira and other remote areas. According to current guidelines, timely identification of HBOC patients may contribute to their ongoing care and treatment.

## Conflicts of interest

The authors declare that they have no conflict of interest.

## Funding declaration

The author(s) received no specific funding for this work.

## Figures and Tables

**Table 1.** Germline pathogenic variants identified in HBOC index patients from non-related families with Madeira ancestry.

	Whole registry	Gene	Variant	Madeira		Whole registry(excluding Madeira)		Ethnicity/Nationality [31]
				Index patients (n)	Variant frequency ¥ (%)	Index patients (n)	Variant frequency^c^ (%)	
***BRCA1*/2**	3,566 patients tested 386 positive index patients	*BRCA1*	c.3331_3334del; p.(Gln1111AsnfsTer5)	6	6/19 (31.6%)	10	10/367 (2.7%)	European, Russian, Colombian, Egyptian, Latin American, Caribbean, Native American
		*BRCA2*	c.156_157insAlu	2	2/19 (10.5%)	86	86/367 (23.4%)	Portuguese founder mutation
			c.298A>T; p.(Lys100Ter)	1	1/19 (5.3%)	0^b^	0	Central/Eastern European
			c.658_659del; p.(Val220IlefsTer4)	4	4/19 (21%)	0^b^	0	Japanese, South-East Poland, Norway, European, African American, Lithuania, Brazil
			c.793+1G>A	1	1/19 (5.3%)	12	12/367 (3.3%)	None specified
			c.9382C>T; p.(Arg3128Ter)	5	5/19 (26.3%)	0^b^	0	Brazil, Norway, Chile; European, African American, Latin
**Other than *BRCA1*/2**	1,185 patients tested 62 positive index patients	*ATM*	c.8264_8268del; p.(Tyr2755CysfsTer12)	1^a^	-	2	2/59 (3.4%)	Spanish, Brazilian
		*PALB2*	c.751C>T; p.(Gln251Ter)	1^a^	-	0^b^	0	Chinese, German and Russian
			c.1633G>T; p.(Glu545Ter)	1	-	0^b^	0	German and Russian
		*RAD50*	c.2516_2517insA; p.(Asp840ArgfsTer5)	1	-	2	2/59 (3.4%)	Latin

a The same patient was diagnosed with both variants

b Variants diagnosed exclusively in the Madeira subgroup

c Number of index patients with specific variant/total of index patients diagnosed with a PV in that cluster (*BRCA1/2* or other than *BRCA1/2*)

## References

[1] Vergote I, Banerjee S, Gerdes AM (2016). Current perspectives on recommendations for BRCA genetic testing in ovarian cancer patients. Eur J Cancer.

[2] Daly MB, Pilarski R, Yurgelun MB (2016). NCCN guidelines insights: genetic/familial high-risk assessment: breast, ovarian, and pancreatic, version 1.2020. J Natl Compr Canc Netw.

[3] Mohler JL, Antonarakis ES, Armstrong AJ (2019). Prostate cancer, version 2.2019, NCCN clinical practice guidelines in oncology. J Natl Compr Canc Netw.

[4] Kemp Z, Turnbull A, Yost S (2019). Evaluation of cancer-based criteria for use in mainstream BRCA1 and BRCA2 genetic testing in patients with breast cancer. JAMA Netw Open.

[5] Kinney AY, Steffen LE, Brumbach BH (2016). Randomized noninferiority trial of telephone delivery of BRCA1/2 genetic counseling compared with in-person counseling: 1-year follow-up. J Clin Oncol.

[6] Zierhut HA, MacFarlane IM, Ahmed Z (2018). Genetic counselors’ experiences and interest in telegenetics and remote counseling. J Genet Couns.

[7] Peixoto A, Santos C, Pinto P (2015). The role of targeted BRCA1/BRCA2 mutation analysis in hereditary breast/ovarian cancer families of Portuguese ancestry. Clin Genet.

[8] Kim H, Choi DH (2013). Distribution of BRCA1 and BRCA2 mutations in Asian patients with breast cancer. J Breast Cancer.

[9] Rafnar T, Benediktsdottir KR, Eldon BJ (2004). BRCA2, but not BRCA1, mutations account for familial ovarian cancer in Iceland: a population-based study. Eur J Cancer.

[10] Peixoto A, Santos C, Rocha P (2009). The c.156_157insAlu BRCA2 rearrangement accounts for more than one-fourth of deleterious BRCA mutations in northern/central Portugal. Breast Cancer Res Treat.

[11] Machado PM, Brandão RD, Cavaco BM (2019). Screening for a BRCA2 rearrangement in high-risk breast/ovarian cancer families: evidence for a founder effect and analysis of the associated phenotypes. J Clin Oncol.

[12] Rebbeck TR, Friebel TM, Friedman E (2018). Mutational spectrum in a worldwide study of 29,700 families with BRCA1 or BRCA2 mutations. Hum Mutat.

[13] Alsop K, Fereday S, Meldrum C (2012). BRCA mutation frequency and patterns of treatment response in BRCA mutation-positive women with ovarian cancer: a report from the Australian Ovarian Cancer Study Group. J Clin Oncol.

[14] Ganguly A, Rock MJ, Prockop DJ (1993). Conformation-sensitive gel electrophoresis for rapid detection of single-base differences in double-stranded PCR products and DNA fragments: evidence for solvent-induced bends in DNA heteroduplexes. Proc Natl Acad Sci U S A.

[15] Mattocks CJ, Watkins G, Ward D (2010). Interlaboratory diagnostic validation of conformation-sensitive capillary electrophoresis for mutation scanning. Clin Chem.

[16] Hill M (2011). Conformation sensitive gel electrophoresis. Methods Mol Biol.

[17] Bexiga C, Nejo P, Oliveira I (2020). Abstract P6-08-17: when *BRCA2*-breast cancer is more prevalent than *BRCA1*-breast cancer: prospective follow-up data from a multidisciplinary program. Cancer Res.

[18] Maistro S, Teixeira N, Encinas G (2016). Germline mutations in *BRCA1* and *BRCA2* in epithelial ovarian cancer patients in Brazil. BMC Cancer.

[19] Alemar B, Herzog J, Netto CBO (2016). Prevalence of Hispanic *BRCA1* and *BRCA2* mutations among hereditary breast and ovarian cancer patients from Brazil reveals differences among Latin American populations. Cancer Genet.

[20] Cotrim DP, Ribeiro ARG, Paixão D (2019). Prevalence of *BRCA1* and *BRCA2* pathogenic and likely pathogenic variants in non-selected ovarian carcinoma patients in Brazil. BMC Cancer.

[21] Rodríguez AO, Llacuachaqui M, Pardo GG (2019). *BRCA1* and *BRCA2* mutations among ovarian cancer patients from Colombia. Gynecol Oncol.

[22] Blay P, Santamaría I, Pitiot AS (2013). Mutational analysis of *BRCA1* and *BRCA2* in hereditary breast and ovarian cancer families from Asturias (Northern Spain). BMC Cancer.

[23] Zhang S, Royer R, Li S (2011). Frequencies of *BRCA1* and *BRCA2* mutations among 1,342 unselected patients with invasive ovarian cancer. Gynecol Oncol.

[24] Damiola F, Pertesi M, Oliver J (2014). Rare key functional domain missense substitutions in MRE11A, RAD50, and NBN contribute to breast cancer susceptibility: results from a Breast Cancer Family Registry case-control mutation-screening study. Breast Cancer Res.

[25] Walsh T, Casadei S, Lee MK (2011). Mutations in 12 genes for inherited ovarian, fallopian tube, and peritoneal carcinoma identified by massively parallel sequencing. Proc Natl Acad Sci USA.

[26] Pinto P, Paulo P, Santos C (2016). Implementation of next-generation sequencing for molecular diagnosis of hereditary breast and ovarian cancer highlights its genetic heterogeneity. Breast Cancer Res Treat.

[27] Bogdanova N, Sokolenko AP, Iyevleva AG (2011). PALB2 mutations in German and Russian patients with bilateral breast cancer. Breast Cancer Res Treat.

[28] Zhang K, Zhou J, Zhu X (2017). Germline mutations of PALB2 gene in a sequential series of Chinese patients with breast cancer. Breast Cancer Res Treat.

[29] Hu C, Hart SN, Gnanaolivu R (2021). A population-based study of genes previously implicated in breast cancer. N Engl J Med.

[30] Dorling L, Carvalho S, Breast Cancer Association Consortium (2021). Breast cancer risk genes – association analysis in more than 113,000 women. N Engl J Med.

[31] https://brcaexchange.org/.

